# Analysis of Global Transcriptome Change in Mouse Embryonic Fibroblasts After dsDNA and dsRNA Viral Mimic Stimulation

**DOI:** 10.3389/fimmu.2019.00836

**Published:** 2019-04-17

**Authors:** Xin Xie, Pu-Ste Liu, Piergiorgio Percipalle

**Affiliations:** ^1^Biology Program, New York University Abu Dhabi (NYUAD), Abu Dhabi, United Arab Emirates; ^2^Institute of Cellular and System Medicine, National Health Research Institutes, Zhunan, Taiwan; ^3^Department of Molecular Biosciences, The Wenner-Gren Institute, Stockholm University, Stockholm, Sweden

**Keywords:** transcriptional profiling, genome-wide analysis, viral mimic stimulation, innate immunity, mouse embryonic fibroblasts

## Abstract

The activation of innate immunity by viral nucleic acids present in the cytoplasm plays an essential role in controlling viral infection in both immune and non-immune cells. The dsDNA and dsRNA viral mimics can stimulate the cytosolic nucleic acids sensors and activate the antiviral innate immunity. In this study, taking advantage of dsDNA and dsRNA viral mimics, we investigated the global transcriptome changes after the antiviral immunity activation in mouse embryonic fibroblasts. Results from our data identified a positive feedback up-regulation of sensors (e.g., *Tlr2, Tlr3, Ddx58, cGAS*), transducers (e.g., *Traf2, Tbk1*) and transcription factors (e.g., *Irf7, Jun, Stat1, Stat2*) in multiple pathways involved in detecting viral or microbial infections upon viral mimic stimulation. A group of genes involved in DNA damage response and DNA repair such as *Parp9, Dtx3l, Rad52* were also up-regulated, implying the involvement of these genes in antiviral immunity. Molecular function analysis further showed that groups of helicase genes (e.g., *Dhx58, Helz2*), nuclease genes (e.g., *Dnase1l3, Rsph10b*), methyltransferase genes (e.g., histone methyltransferase *Prdm9, Setdb2*; RNA methyltransferase *Mettl3, Mttl14*), and protein ubiquitin-ligase genes (e.g., *Trim* genes and *Rnf* genes) were up-regulated upon antiviral immunity activation. In contrast, viral mimic stimulation down-regulated genes involved in a broad range of general biological processes (e.g., cell division, metabolism), cellular components (e.g., mitochondria and ribosome), and molecular functions (e.g., cell-cell adhesion, microtubule binding). In summary, our study provides valuable information about the global transcriptome changes upon antiviral immunity activation. The identification of novel groups of genes up-regulated upon antiviral immunity activation serves as useful resource for mining new antiviral sensors and effectors.

## Introduction

Antiviral innate immunity serves as a primary barrier to control viral infection in both immune and non-immune cells before the development of sophisticated adaptive immunity. Previous studies unveiled several pathways that are involved in sensing viral infection and mounting the subsequent antiviral immunity. Toll-like receptors (TLRs) 3, 7, 8, and 9 are a group of pattern recognition receptors (PRRs) detecting early viral infection events in endosome (1). In the cytoplasm, dsRNA or 5′triphosphate-modified RNA derived from virus can be sensed by RIG-I like receptors (RLRs), such as RIG-I, MDA5, LGP2 (2, 3). Cytosolic DNA sensors such as cGAS, DAI and AIM2 are responsible for the detection of viral dsDNA (4). The activation of these nucleic acids sensors recruits and activates different adaptors for signal transduction. For example, TLRs except for TLR3 utilize MyD88 as an adaptor molecule to recruit downstream signaling transducers including protein kinases IRAK4 and IRAK1, and the ubiquitin ligase TRAF6 (5). Upon the binding of dsRNA, RIG-I and MDA5 leads to the activation and polymerization of mitochondrial membrane protein MAVS for signal amplification (6). The signal transduction from the activation of TLRs or RLRs eventually activates the protein kinase TBK1, which phosphorylates and activates transcription factors IRF3 and IRF7 to promote the production of type I interferons (7). The secretion of type I interferons such as IFN-α and IFN-β, acts in an autocrine or paracrine manner to stimulate the expression of interferon-stimulated genes (ISGs) which exhibit anti-proliferative and antiviral effects (8).

Recent studies further identified more genes with RNA and DNA sensing ability, which may serve as sensors for viral infection. Nod2, a previously identified bacterial peptidoglycan sensor (9), can also detect the RNA genome of virus and activate IRF3 to produce type I interferon (10). DDX41 has been proposed to be a cytosolic DNA sensor as it has been shown to bind dsDNA and associate with STING and TBK1 (11). Interestingly, some genes involved in DNA damage response and repair were also found to have cytosolic DNA sensing activity, such as DNA-PK (DNA-dependent protein kinase) (12) and Rad50 (13). It is anticipated that novel cytosolic RNA and DNA sensors remain to be discovered by future studies.

Antiviral innate immunity is dynamically regulated by cellular ubiquitin system. For certain virus, ubiquitination of viral proteins can promote their degradation and restrict viral infection (14, 15). The ubiquitination also controls the activity of sensors or effectors in antiviral immunity. For example, the activation of RNA sensor RIG-I depends on its ubiquitination, which can be mediated by ubiquitin ligases Trim4, Trim25, and Rnf135 (16–19). Trim6 and Herc6 can ubiquitinate other antiviral effectors to modulate their activity (20, 21). In contrast, Trim29 negatively regulates RIG-I-mediated innate immune response and targets DNA sensor STING for degradation (11, 22, 23). Therefore, multiple ubiquitin-transferases or ligases serves as a layer of post-translational regulation of antiviral innate immunity.

Poly(deoxyadenylic-deoxythymidylic) acid [poly(dA-dT)] and Polyinosinic-polycytidylic acid [poly(I:C)] are widely used viral nucleic acids analogs in antiviral immune responses (24–27). poly(dA-dT) is recognized by several cytosolic DNA sensors including ZBP1/DAI and cGAS (25, 26). The synthetic dsRNA analog poly(I:C) binds and activates RNA sensors such as RIG-I and MDA-5 (27). RIG-I can also sense poly(dA-dT) indirectly by recognizing the dsRNA with a 5′-triphosphate derived from poly(dA-dT) (24). These viral mimics have been shown to activate the antiviral immune responses and induce the expression of type I interferon in a variety of mammalian cells, including mouse embryonic fibroblasts, human epithelial cells, rat hepatocyte, dog keratinocyte (28–31).

In this study, we are mainly investigating the global transcriptome changes after the antiviral immunity activation in mouse embryonic fibroblasts, with the aim to identify novel biological processes, molecular functions and pathways being up or down-regulated at transcription level. We stimulated cells with poly(dA-dT) and poly(I:C) to mimic dsDNA and dsRNA virus, and analyzed the genes commonly affected by both viral mimics. Our analysis first demonstrated a widespread positive feedback up-regulation of sensors, transducers, and transcription factors in multiple pathways involved in detecting viral or microbial infections, suggesting a self-enhancement of antiviral pathways at transcription level upon viral infection. Viral mimic stimulation also up-regulated a group of genes involved in DNA damage response and DNA repair, implying the involvement of these genes in antiviral immunity. Molecular function analysis further showed that groups of helicase genes, nuclease genes, methyltransferase genes and protein ubiquitin-ligase genes were up-regulated upon antiviral immunity activation. Some of those genes have been characterized as antiviral sensors, effectors or regulators by previous studies, while the potential function of many of those genes in antiviral immunity remain to be investigated. In contrast, viral mimic stimulation down-regulated genes involved in a wide spectrum of general biological processes, cellular components and molecular functions. Taken together, our study provides valuable information about the global transcriptome changes upon the activation of antiviral immunity. We identified novel groups of genes being up-regulated upon antiviral immunity activation, which serves as useful resources for mining new antiviral sensors and effectors.

## Materials and Methods

### Cell Culture

The mouse embryonic fibroblasts (MEFs) (Wilde type mouse embryonic fibroblast from the lab of Dr. Christophe Ampe, University of Gent, Belgium) (32) were maintained and cultured with Dulbecco's modified Eagle medium (DMEM) with high glucose (Sigma, D5671), 10% fetal bovine serum (Sigma, F0804), and 100 units/mL penicillin and 100 μg/mL streptomycin (Sigma, P4333), in a humidified incubator with 5% CO^2^ at 37°C.

### Viral Mimic Stimulation

MEFs were seeded at 5 × 10^5^ cells/well in 6-well plates the day before treatment. Poly (I:C) LMW (InvivoGen, tlrl-picw) and Poly (dA:dT) naked (InvivoGen, tlrl-patn) were re-constituted in sterile/endotoxin-free physiological water (InvivoGen, tlrl-phy10). Transfection was performed using Lipofectamine 2000 reagent (Invitrogen, 11668019). Fifteen micrograms Poly (I:C) or 15 μg Poly (dA:dT) was mixed with 9 μL Lipofectamine 2000 reagent in 250 μL Opti-MEM™ I reduced serum medium (Gibco, 31985070) and incubated for 10 min. The culture medium in 6 well plate were replaced with 1.25 mL Opti-MEM™ I reduced serum medium and the Poly (I:C) or Poly (dA:dT) transfection mixture were added to the cells (Final concentration for both Poly (I:C) or Poly (dA:dT) is 10 μg/ml). For the mock group, medium mixed with 9 μL Lipofectamine 2000 only was added to the cells. Cells were further cultured in the incubator for 6 h before total RNA extraction for both mock and treatment groups. For each condition, 3 biological replicates were prepared for RNA isolation.

### RNA Library Construction and Sequencing

Three biological replicates of each experimental group were prepared for RNA-seq analysis. Briefly, cells in mock or treatment group were washed once with cold PBS and then total RNA were purified using RNeasy Mini Kit (Qiagen, 74106) according to the manufacturer's instruction. RNA-sequencing libraries were prepared using TruSeq RNA Library Prep Kit v2 (Illumina, RS-122-2002). Briefly, total RNA was mixed with magnetic Oligo-dT beads to purify the mRNA. Then the purified mRNA on beads were fragmented and primed for cDNA synthesis. The first strand and second strand cDNA synthesis was performed using SuperScript™ double-stranded cDNA synthesis kit (Invitrogen, 11917020) with random primers. The cDNA were then purified using AMPure XP Beads (Beckman Coulter, A63881). The cDNA was end-repaired and adenylated at 3′ end, followed by the ligation of the adaptors. The cDNA was then amplified with index primers using the following protocol: 98°C 30 s; 15 cycles of: 98°C 10 s, 60°C 30 s, and 72°C 30 s; 72°C 5 min. The PCR product was purified using AMPure XP Beads and the library quality and size was analyzed using 2100 Bioanalyzer (Agilent Genomics). Libraries with compatible index primers were pooled at equal amount and deep-sequencing was performed using Illumina HiSEq 2500 sequencing platform (New York University Abu Dhabi Sequencing Center). RNA-seq data were deposited in GEO database: Accession number: GSE111938.

### RNA-Seq Data Analysis

The data was processed through the standard RNAseq analysis pipeline at NYUAD. Briefly, raw read alignment was performed using tophat2 v2.1.0, with the parameters “–no-novel-junctions” and “–G” when specifying the genome file. The reference genome and GFF annotation correspond to the *Mus musculus* GRCm38.p4 genome version. Following the tophat2 alignment, read counts mapped to each gene were generated using HTseq count (33). The differential expression analysis of the raw counts were performed based on the DESeq2 R library (34). The START Web-based RNA-seq analysis and visualization resources (35) was used to perform the differential expression test and visualization. FDR-adjusted *p*-value after Benjamini–Hochberg correction for multiple-testing were used as the statistics to define the differential expression. Genes with FDR-adjusted *p* < 0.05 are considered to be significantly differentially expressed between two samples.

### Gene Ontology Enrichment and KEGG Pathway Analysis

Genes with FDR-adjusted *p* < 0.05 were considered to be differentially expressed between Poly(I:C) vs. Mock group or Poly(dA:dT) vs. Mock group. The commonly up-regulated and down-regulated genes in Poly(I:C) group and Poly(dA:dT) group in comparison to Mock group were submitted to gene ontology (GO) enrichment and KEGG pathway analysis using the Web-based DAVID bioinformatics resources 6.8 (36). For the GO terms or KEGG pathway terms to be considered as over-represented or enriched in each gene list, the following criteria was applied: 1. ≥20 genes were found to be associated with the GO term or KEGG term in the database; 2. The test *p*-value [a modified Fisher Exact Test *P*-value (EASE Score): the smaller, the more enriched] is < 0.01; 3. The fold of enrichment (observed number of genes in the term/expected number of genes in the term) is ≥1.5. The GO terms or KEGG pathway terms that were observed exclusively in up-regulated or down-regulated gene list were further analyzed in details.

### Quantitative Real-Time qPCR Analysis

Total RNA was extracted using RNeasy Mini Kit (Qiagen) according to the manufacturer's instructions. RNA was reverse transcribed to cDNA by RevertAid First Strand cDNA synthesis Kit (Thermo Fisher Scientific), based on the manufacturer's instructions. Diluted cDNA was subjected to quantitative real-time PCR analysis using Maxima SYBR Green qPCR Mix (Thermo Fisher Scientific) on StepOne Plus Real-Time PCR system (Applied Biosystems). All the target gene expression level was normalized to the expression of *Nono* reference gene. Primers of qPCR were listed in Table S1.

## Results

### Viral Mimic Stimulation Leads to Skewed Distribution of Gene Expression Change in Mouse Embryonic Fibroblasts

**Figure 1:**
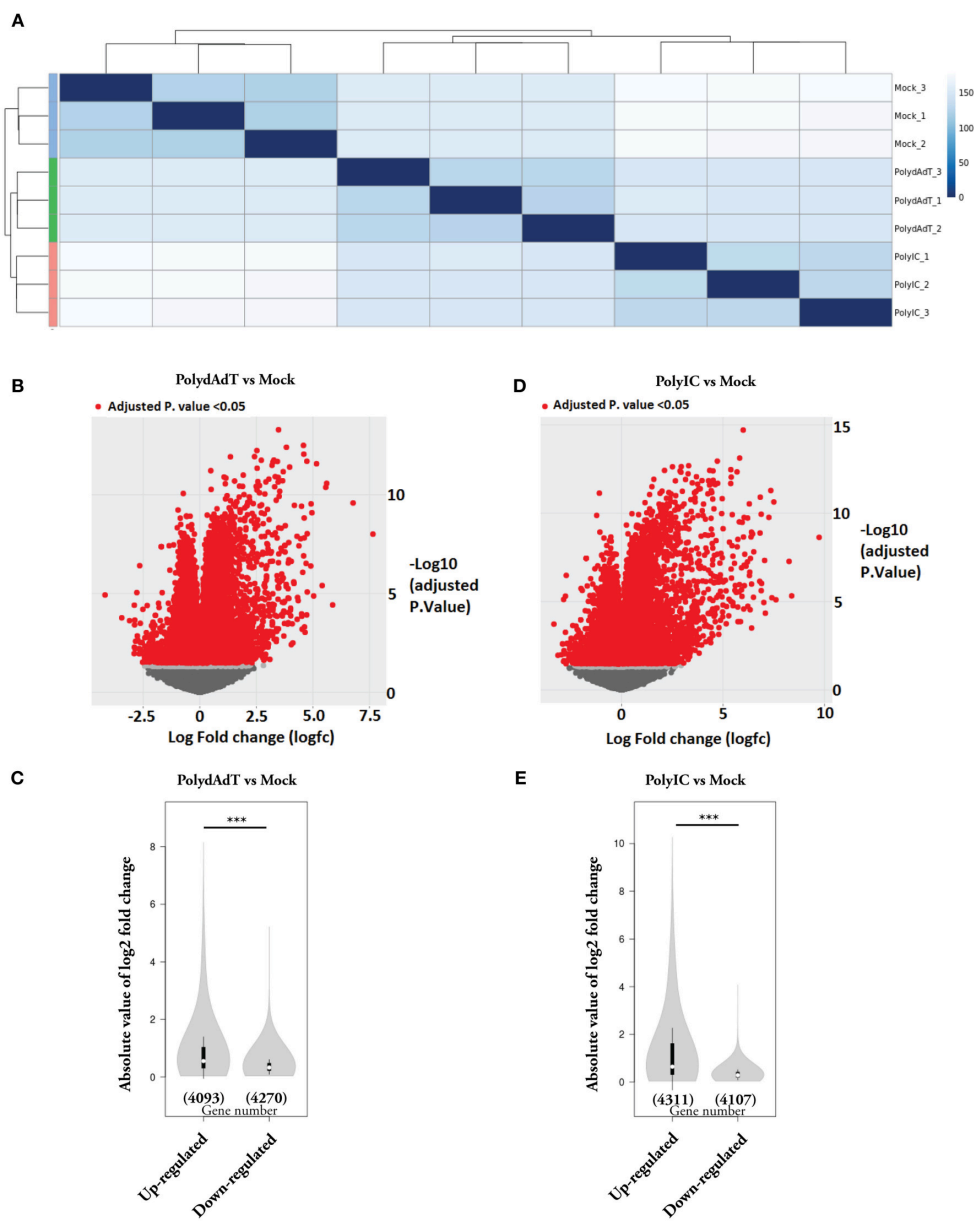
Gene expression changes caused by viral mimic stimulation. **(A)** Quantification of the similarity in gene expression profiles of MEFs stimulated by poly(dA:dT) or poly(I:C) for 6 h. Mock groups: treated with transfection reagent alone. Euclidean distances were calculated from normalized log-transformed read counts in three biological replicates of each group. **(B)** Volcano plot showing the relative expression of genes in PolydAdT group vs. Mock group. X-axis shows the log (fold change), Y axis is the FDR-adjusted *p*-value. Genes with FDR-adjusted *p*-value < 0.05 was considered to be differentially expressed (Red dots). **(C)** Violin plot of the absolute log2 (fold change) of up-regulated or down-regulated genes in PolydAdT group vs. Mock group. **(D)** Volcano plot showing the relative expression of genes in PolyIC group vs. Mock group. **(E)** Violin plot of the absolute log2 (fold change) of differentially expressed genes in PolyIC group vs. Mock group. Statistics in **(C,E)**: Mann–Whitney *U*-test, ***p < 0.001.

In this study, we investigated the global transcriptome changes in response to viral infection in non-immune cells. We applied poly(deoxyadenylic-deoxythymidylic) acid [poly(dA-dT)] and Polyinosinic-polycytidylic acid [poly(I:C)] to mouse embryonic fibroblast (MEFs). Both poly(dA-dT) and (poly(I:C) are pathogen associated molecular pattern associated with dsDNA and dsRNA viral infection (37, 38). After 6 h treatment with poly(dA-dT), poly(I:C) and mock solution containing transfection reagent alone, we isolated RNA from three biological replicates in each condition and subjected it to RNA-seq analysis. Hierarchical clustering analysis showed that biological replicates in each condition were grouped together and samples stimulated with poly(dA-dT) and poly(I:C) were clustered together (Figure 1A). We next compared the transcriptomes of poly(dA-dT)- and poly(I:C)-treated cells with those from the Mock group. Genes with false discovery rate (FDR)-adjusted *p* < 0.05 were considered as differentially expressed (Figures 1B,D, red dots). We observed a highly skewed distribution of differentially expressed (DE) genes in both poly(dA-dT) and poly(I:C) treated groups, in comparison to mock group. The overall fold change of genes being up-regulated were significantly higher than that of genes being down-regulated (Figures 1B–E). However, the number of genes being down-regulated was approximately equal to that of the up-regulate ones (Figures 1C,E). These data altogether suggests that viral infection can lead to both induction and suppression of genes. The extent of gene induction is significantly higher than that of gene down-regulation.

### Different Sets of Gene Programs Are Up-Regulated or Down-Regulated After Viral Mimic Stimulation

**Figure 2:**
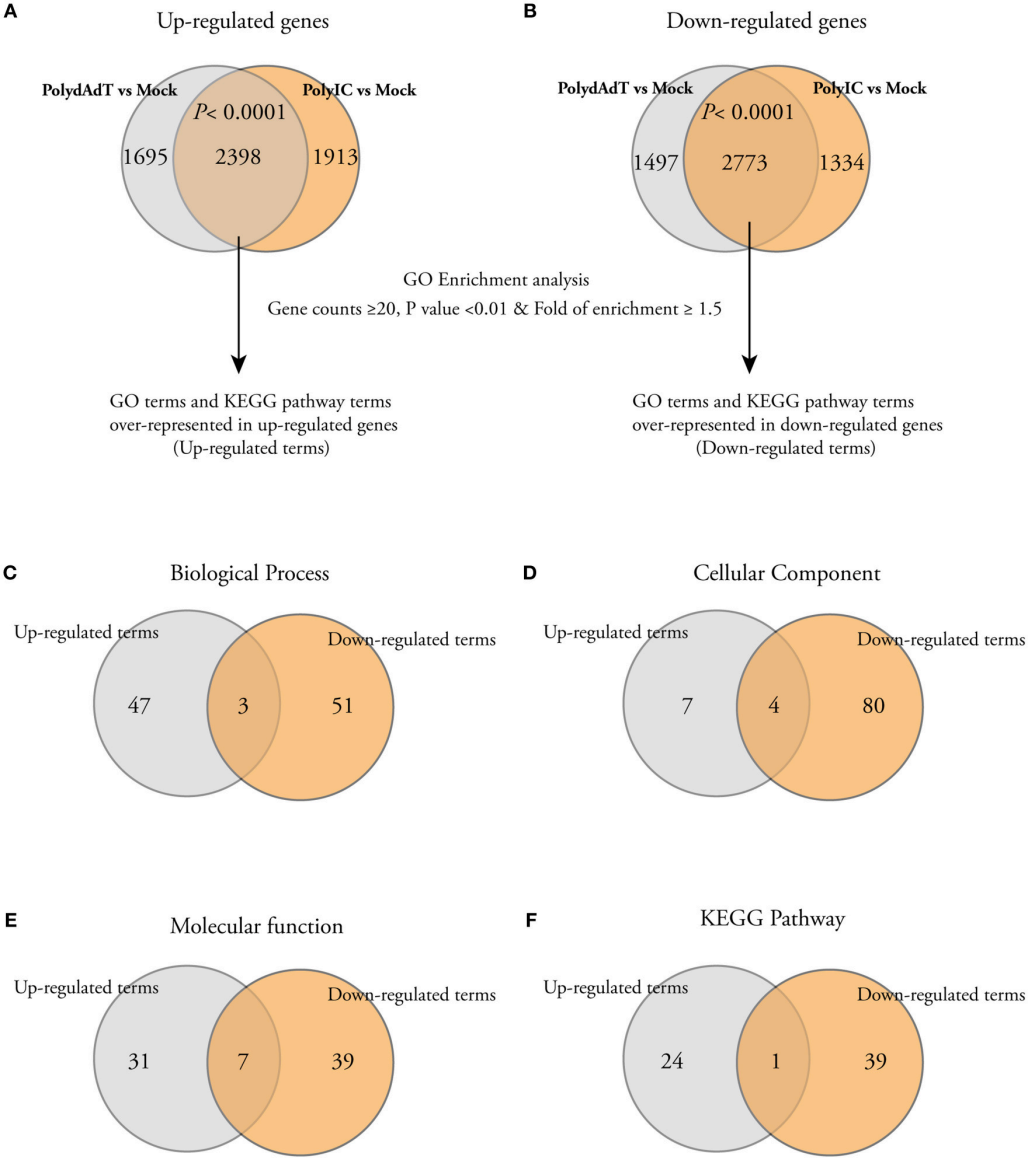
Gene Ontology (GO) and KEGG pathway analysis show that different cellular processes, components, and pathways are enriched among both up-regulated and down-regulated genes. **(A,B)** Venn diagrams showing that a significant number of genes is commonly up-regulated or down-regulated by PolydAdT and PolyIC: Fisher's exact test. The commonly up-regulated or down-regulated genes were subject to gene ontology enrichment analysis and KEGG pathway analysis using DAVID bioinformatics resources. The GO terms or KEGG terms with at least 20 gene counts, *p* < 0.01 and the fold of enrichment ≥1.5 were considered to be over-represented in each gene list. **(C–F)** Venn diagrams shows that the majority of over-represented GO and KEGG terms in biological process **(C)**, cellular component **(D)**, molecular function **(E)**, and KEGG pathway **(F)** are differentially enriched in up-regulated and down-regulated genes.

In order to get biological insights about the up-regulated and down-regulated genes, we performed Gene Ontology (GO) enrichment analysis. We first isolated the genes commonly up-regulated or down-regulated by poly(dA-dT) and poly(I:C) because they are representatives of the genes potentially affected by both dsDNA and dsRNA viral infection. There was a highly significant overlap of up-regulated or down-regulated genes between poly(dA-dT) and poly(I:C) treatment groups (Figures 2A,B), indicating that the dsDNA and dsRNA viral infection induce common transcriptome changes. We then used the commonly up-regulated or down-regulated genes to perform GO enrichment analysis separately. The GO terms or the KEGG pathway terms with gene counts ≥20, *p* < 0.01 and fold of enrichment ≥1.5 were considered to be significantly over-represented in the gene list (Figures 2A,B). The full list of significantly up-regulated or down-regulated GO terms and KEGG pathway terms can be found in Table S2. When we compared the enriched terms in each category (Biological process, Cellular component, Molecular function, and KEGG pathway), the majority of them were not shared by the up-regulated and down-regulated genes (Figures 2C–F). These data shows that after viral mimic stimulation, there are gene programs associated with different biological processes, cellular components, molecular functions, and signaling pathways that can be up-regulated or down-regulated, respectively.

### Activation of Antiviral Immunity Leads to a Positive Feedback Regulation of Multiple Signaling Pathways Involved in Sensing Viral or Microbial Infection

**Figure 3:**
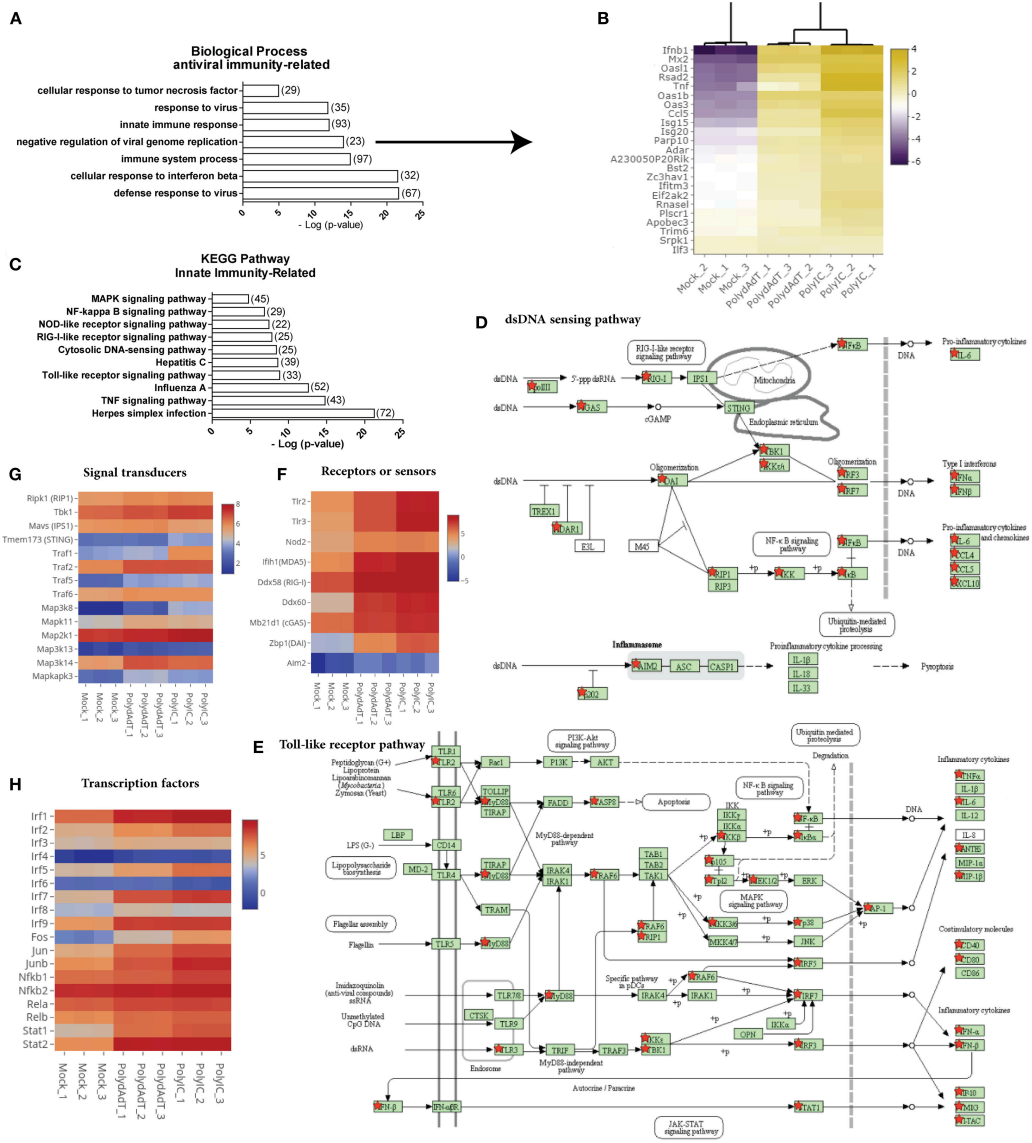
Stimulation by viral mimics up-regulates genes involved in multiple innate immunity pathways. **(A)** Top biological processes related to antiviral immunity over-represented in up-regulated genes. Number in bracket indicates the number of genes associated with the term. **(B)** Heat map showing the genes involved in the negative regulation of viral genome replication are commonly up-regulated by PolydAdT and PolyIC stimulation. Scale bar: log2 (relative expression level). **(C)** Innate immunity pathways over-represented in the up-regulated genes. **(D,E)** Up-regulated genes that are mapped to the dsDNA sensing pathway **(D)** and Toll-like receptor pathway **(E)** using KEGG pathway database, which are marked by red stars. **(F–H)** The relative expressions of genes serving as receptors or sensors **(F)**, signaling transducers **(G)**, and transcription factors **(H)** in innate immunity pathways. Scale bar: log2 (relative expression level). Pathway maps in **(D,E)** are modified from KEGG pathway maps (with copyright permission for publication) using DAVID bioinformatics tool (36).

We next examined the biological processes enriched in the commonly up-regulated genes. As expected, anti-viral innate immunity processes such as defense response to virus, cellular response to interferon beta, negative regulation of viral genome replication and innate immune response were highly over-represented in the up-regulated genes (Figure 3A). For example, genes involved in negative regulation of viral replication such as *Mx2, Isg15, Isg20, Rnasel*, and *Oas* family genes were highly up-regulated after viral mimic stimulation (Figure 3B) (39–43), as well as antiviral cytokine genes *Ifnb1* and *Tnf* (44). Different classes of genes in defense response to viruses were also highly induced (Figure S1). These data demonstrates that the viral mimic stimulation of embryonic fibroblasts with poly(dA-dT) and poly(I:C) faithfully activates the antiviral immunity responses.

When we examined the KEGG pathway terms enriched in the up-regulated genes, we found that multiple innate immunity signaling pathways involved in sensing virus or microbe infection were significantly over-represented. These includes Toll-like receptor signaling pathway, cytosolic DNA sensing pathway, RIG-I-Like receptor signaling pathway, NOD-like receptor pathway (Figure 3C). When we mapped the up-regulated genes to individual pathways, we found that not only the target genes induced by these pathways were up-regulated, multiple components functioning as receptors, signaling transducers or transcription factors at different stages of those pathways were also up-regulated (Figures 3D,E; Figures S2A,B). For example, Toll-like receptors (*Tlr2, Tlr3*), RNA sensors (*Ifih1, Ddx58, Nod2*), and DNA sensors (*Mb21d1, Ddx60, Zbp1*, and *Aim2*) which are functioning in different pathways were up-regulated to different levels (Figure 3F). The up-regulated signaling transducers includes kinases such as *Ripk1, Tbk1*, kinases in MAPK (Mitogen activated protein kinase) signaling pathways and several TRAF (TNF receptor associated factor) members (Figure 3G). Transcription factors such as several members in IRF (Interferon-regulatory factor) family, AP-1 (Activating protein 1) family, NF-κB (Nuclear factor-kappaB) family, and STAT (signal transducer and activator of transcription) family were also highly induced (Figure 3H). Collectively, these results indicate that the activation of antiviral immunity response leads to a widespread positive feedback regulation of multiple signaling pathways involved in detecting viral or microbial infections. The positive feedback enhances the expression of genes functioning at different steps of multiple signaling pathways.

### Antiviral Immunity Activation Up-Regulates Genes in DNA Damage Response and DNA Repair

**Figure 4:**
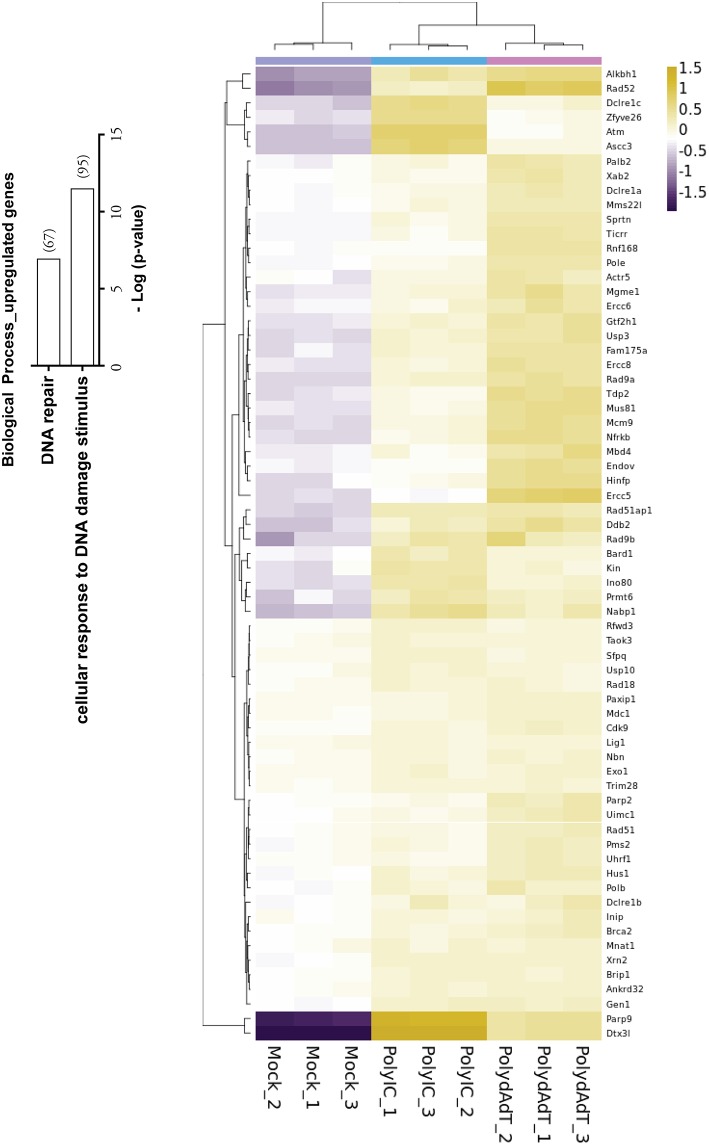
Histogram showing the –Log (*p*-value) of the enrichment of genes in biological processes of DNA repair and cellular response to DNA damage stimulus, with the number of genes up-regulated in bracket. Heat map shows the genes in DNA repair process that are up-regulated by PolydAdT and PolyIC stimulation.

Recent studies have shown that viral infection can lead to the activation of signaling processes of DNA damage responses at protein level in the host cells (45, 46). Our data also revealed that biological processes such as cellular response to DNA damage stimulus and DNA repair were significantly enriched in the up-regulated genes (Figure 4). Those genes includes *Atm, Brca2*, several members in PARP (Poly (ADP-ribose) polymerase) family and DNA repair proteins *Rad9b, Rad18, Rad 51, Rad52* (Figure 4). Interestingly, the two heavily induced genes *Parp9* and *Dtx3l* are well characterized DNA damage response proteins that form a heterodimer in DNA repair (47, 48). Parp9 and Dtx3l have been recently implicated in enhancing interferon signaling and controlling viral infection (49). This study and our data altogether suggest that DNA damage repair genes being up-regulated upon viral infection may regulate the antiviral immunity signaling process. Consistently, here we report the identification of a large group of DNA repair genes that can be induced upon antiviral immunity activation, providing a valuable resource for future investigation of their potential roles in viral infection defense.

### Molecular Function Analysis Reveals Induction of Helicases, Nucleases, Methyltransferases, and Protein Ubiquitin-Ligases Upon Antiviral Immunity Activation

**Figure 5:**
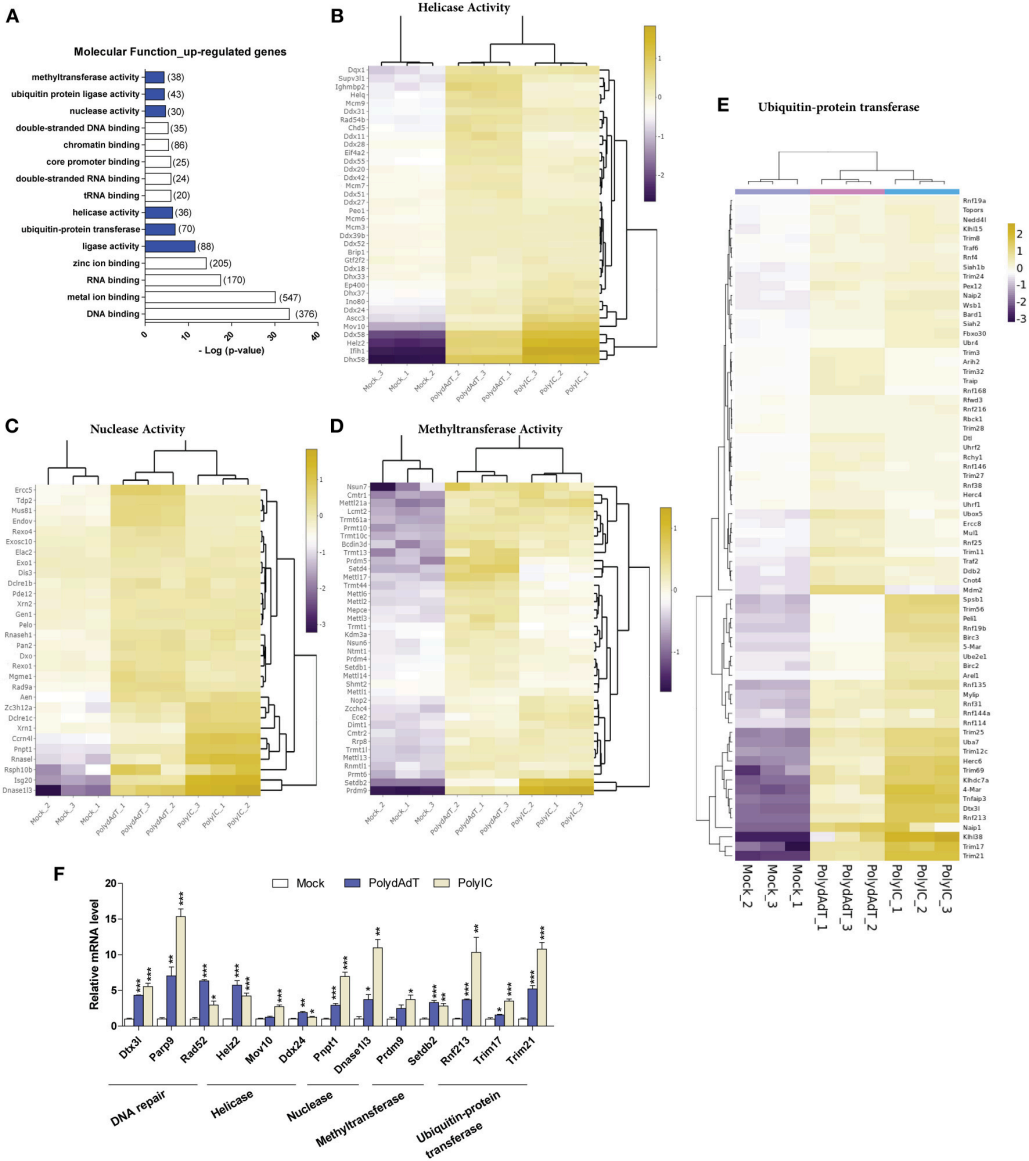
Viral mimics stimulation up-regulates genes with enzymatic activity in DNA and RNA processing. **(A)** Top molecular functions over-represented in the up-regulated genes. Number in bracket indicates the number of genes associated with the term. **(B–E)** Heat maps showing genes with helicase activity **(B)**, nuclease activity **(C)**, methyltransferase activity **(D)**, and ubiquitin-protein transferase **(E)** that are up-regulated by viral mimic stimulation. Scale bar: log2 (relative expression level). **(F)** qPCR quantification of the relative expression of selected genes of different molecular functions. *Nono* housekeeping gene was used as the internal control for normalization. Student's t-test was applied to compare the value of PolydAdT or PolyIC to the Mock group respectively. *p < 0.05, **p < 0.01, ***p < 0.001. *n* ≥ 3 biological replicates.

To investigate whether the up-regulated genes are related to certain molecular functions, among the enriched GO terms we examined molecular functions. We found that except for DNA or RNA binding functions, genes which encode proteins with different enzymatic activities were found to be enriched within the up-regulated genes. These molecular functions include ligase activity, helicase activity, nuclease activity, methyltransferase activity as well as ubiquitin-protein ligase and transferase activity (Figure 5A). For the helicase activity, among the most heavily induced genes we found *Ddx58* (RIG-I), *Ifih1* (MDA5), and *Dhx58* (LGP2), which are well-characterized viral sensors in innate immunity (50). The recently identified antiviral effectors *Mov10* (51) and *Helz2* (52) were also highly up-regulated (Figure 5B). Apart from that, multiple members in MCM (Minichromosome maintenance protein complex) family, DDX (DEAD-box helicases) family, DHX (DEAH-box helicases) family were significantly induced upon antiviral immunity activation (Figure 5B). In addition, helicase components of certain chromatin remodeling or modifying complexes such as *Ino80, Chd5, Ep400, Ascc3* were also induced (Figure 5B). These results show that a variety of helicases (including the ones with unclear roles in viral defense such as MCM complex members) are up-regulated, suggesting a diverse group of helicases may be induced and function in antiviral defense upon viral infection.

Consistent with previous studies, the nucleases that are known to be induced by viral infection or interferon such as *Isg20* (40), *Pnpt1* (53), *Rnasel* (54), and *Zc3h12a* (55) were found to be heavily up-regulated in our data sets (Figure 5C). Our data further identified numerous ribonuclease and deoxyribonuclease genes with unknown roles in viral infection such as *Dnase1l3, Dclre1c, Rexo1, Dxo*, and *Mgme1* (Figure 5C). Interestingly, the up-regulated nucleases seem to play diverse functional roles in different cellular processes. For example, Mgme1 exonuclease is required for mitochondrial genome synthesis (56), Dnase1l3 is involved in degrading chromatin released by apoptotic cells (57), and Dclre1c has a functional role in DNA damage repair (58). Therefore, our data suggest that a variety of nuclease genes can be induced by the activation of antiviral immunity and they may be involved in restricting the viral genome replication.

Recent studies identified novel roles of certain histone methyltransferases in antiviral immunity. For example, Setdb2 and Prmt6 were involved in defense against Influenza A virus and HIV-1 infection (59, 60). Our data also showed that the methyltransferase activity was significantly over-represented in the up-regulated genes upon antiviral immunity activation (Figure 5A). Among the up-regulated methyltransferase genes we found histone methyltransferases such as *Setdb1, Setdb2, Setd4, Prmt6, Prdm4*, and *Prdm9* (Figure 5D). Surprisingly, a lot of genes with RNA methyltransferase activity were also induced. Multiple members of Mettl (Methyltransferase like) gene family showed significant up-regulation, such as *Mettl1, Mettl2, Mettl3, Mettl6, Mettl13*, and *Mettl14* (Figure 5D). Other RNA-related methyltransferases included those modifying rRNA (Rnmtl1, Dimt1, Nop2), tRNA (Trmt1l, Trmt1, Trmt13, Trmt10c, Trmt44), or methylating the mRNA 5′-Cap (Cmtr1, Cmtr2) (Figure 5D). Altogether, these findings show that multiple genes related to histone and RNA methyltransferase activity are up-regulated upon antiviral immunity activation, suggesting novel functions of those methyltransferases in controlling viral genome or transcripts after viral infection.

The above results show that antiviral immunity activation lead to the increased expression of helicases, nucleases and methyltransferases which may potentially modify the viral DNA or RNA during infection. At protein level, recent studies have begun to reveal that the ubiquitin-proteasome system plays an important and complex role during viral infection (61–63). Our RNA-seq data analysis also identified ubiquitin-protein transferase and ligase activity as molecular functions that were significantly over-represented in the up-regulated genes after viral mimic stimulation (Figure 5A). The up-regulated genes with ubiquitin-protein transferase activity contain multiple members of Trim (Tripartite motif containing) gene family and Rnf (Ring finger proteins) gene family (Figure 5E). Some of those ubiquitin ligase genes are reported to have antiviral functions. For example, Trim6 and Herc6 were found to potentiate the antiviral immunity signaling pathway (20, 21). However, the antiviral activity of Trim56 seems not to be attributed to the augmentation of interferon antiviral response (64). Whether the other Trim and Rnf genes have similar functions in regulating antiviral immunity remains unclear. We speculate that these up-regulated ubiquitin transferase genes may function to modify the antiviral signaling processes or directly target viral proteins.

Collectively, we discovered that numerous genes with helicase, nuclease, methyltransferase, ubiquitin-transferase functions are up-regulated upon viral mimic stimulations. We also verified the up-regulation of selected genes from different molecular functions by qPCR. The analysis shows that nearly all of these genes display significant induction upon PolydAdT and PolyIC stimulation (Figure 5F). Some of those genes have been reported to have antiviral activity or play roles in antiviral signaling processes. However, the potential roles of the majority of those genes in antiviral immunity remains to be further investigated.

### Viral Mimic Stimulation Down-Regulates Genes Involved in a Wide Spectrum of General Biological Processes, Cellular Components, and Molecular Functions

**Figure 6:**
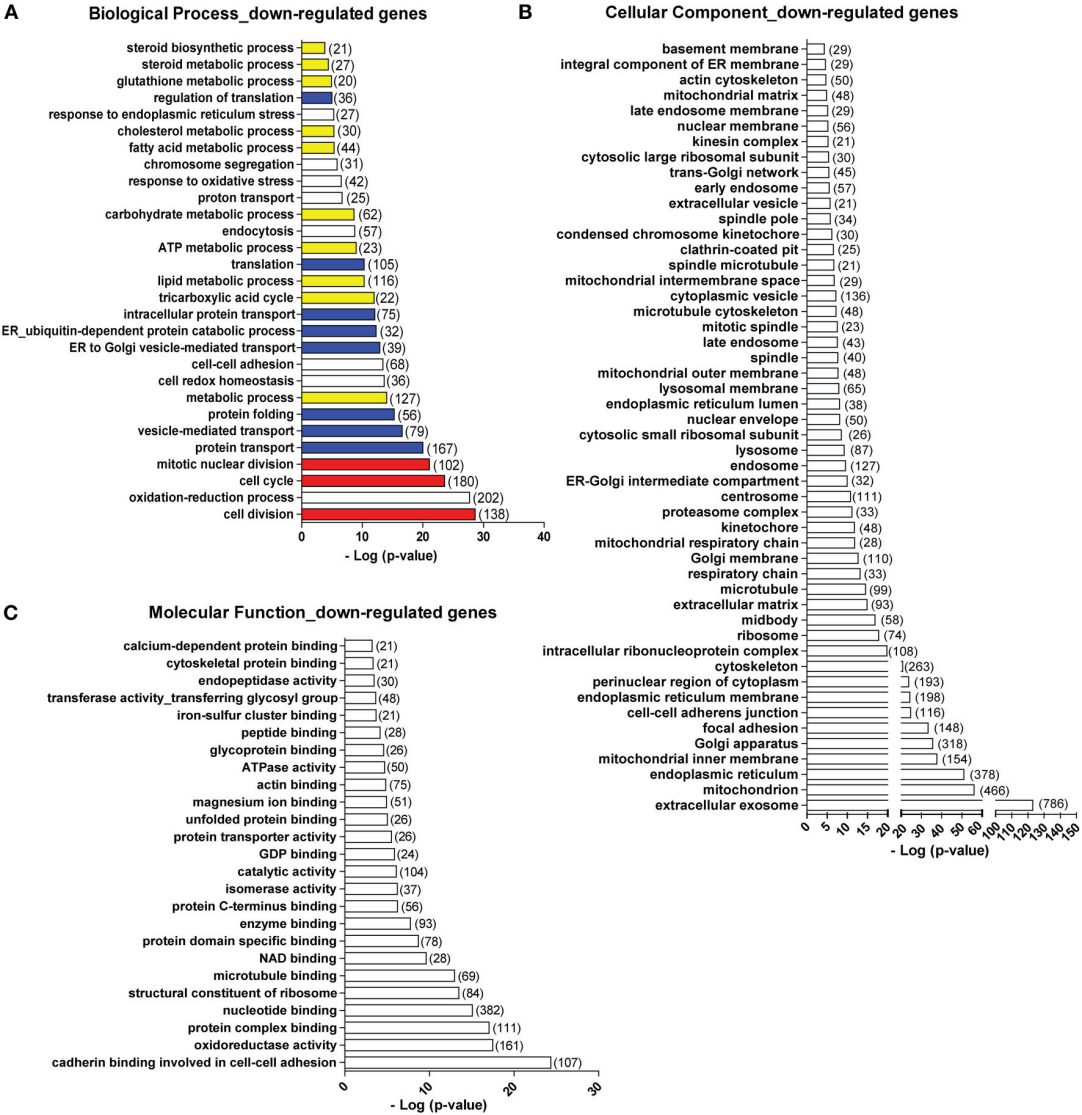
Top biological processes **(A)**, cellular components **(B)**, and molecular functions **(C)** over-represented in the down-regulated genes. Number in bracket indicates the number of genes associated with the term. Colors in **(A)**: red, cell cycle-related terms. Blue: protein translation and processing-related terms. Yellow: metabolic process-related terms.

Apart from those gene programs being up-regulated, we found that following viral mimic stimulation there are many gene programs that are specifically over-represented among the down-regulated genes (Figure 2). Here, we only examined the gene ontology terms uniquely over-represented in the down-regulated gene list. The biological processes significantly enriched in the down-regulated genes include cell cycle and division (in red), protein translation, folding and transport (in blue), and a variety of metabolic processes (in yellow) (Figure 6A). In terms of cellular components, genes associated with almost all cellular components showed down-regulations. The most significantly affected cellular components were extracellular exosome (with 786 genes down-regulated), mitochondrion (with 466 genes down-regulated), endoplasmic reticulum (with 378 gene down-regulated), Golgi apparatus (with 318 genes down-regulated) (Figure 6B). Actin and microtubule cytoskeleton, ribosome, lysosome, extracellular matrix, proteasome complex, spindle were also identified among the down-regulated genes (Figure 6B). For molecular functions, the most significantly affected ones were cadherin binding involved in cell-cell adhesion, oxidative reductase activity, structural constituent of ribosome, microtubule binding (Figure 6C). We also analyzed the down-regulated genes using KEGG pathway database. Pathways related to cell cycle, protein processing in ER, multiple metabolic pathways, oxidative phosphorylation in mitochondria and ribosome were significantly over-represented in the down-regulated genes (Figure S3A), which is highly consistent with the biological processes and cellular components. When we mapped the down-regulated genes to each pathway, we found that the down-regulated genes were situated at different parts of each pathway (Figures S3B, S4, down-regulated genes are labeled with red asterisk). For example, in cell cycle, different cyclin-dependent kinases (CDKs) are down-regulated (Figures S4A, S5A). In Citrate cycle (TCA cycle), genes involved in almost every step of this metabolic pathway were affected (Figures S4B, S5B). In endoplasmic reticulum, multiple genes involved in protein folding and degradations are affected (Figures S4C, S5C).

**Figure 7:**
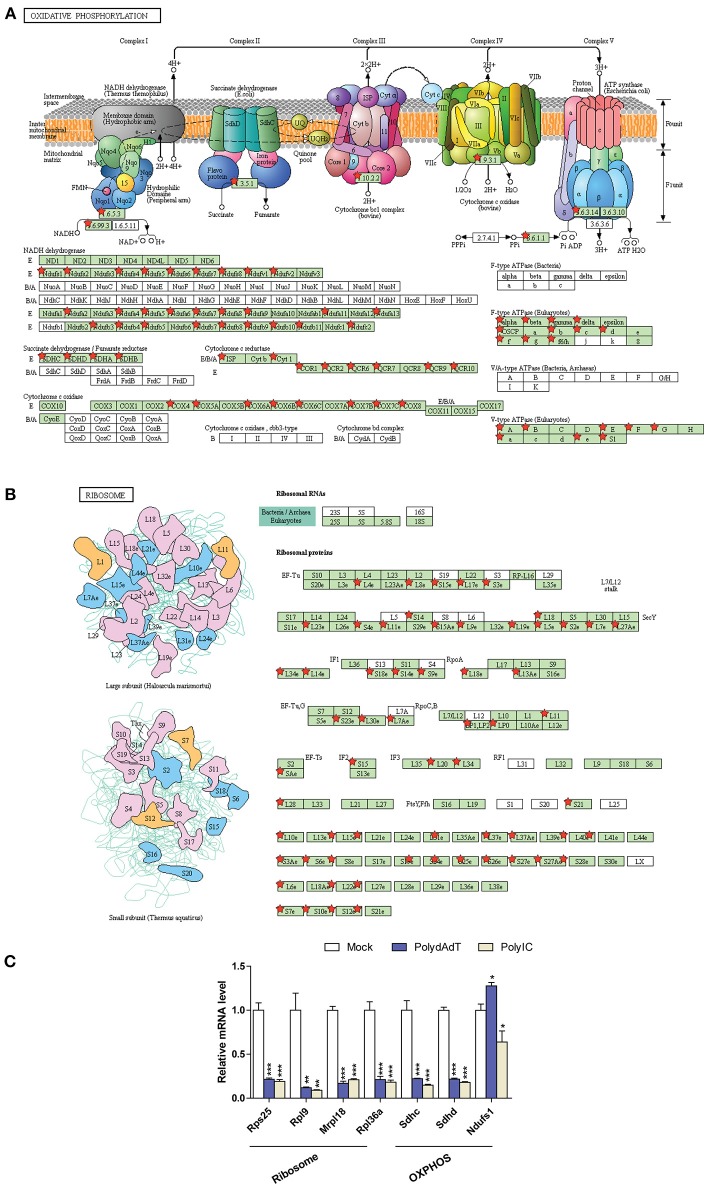
Down-regulated genes associated with oxidative phosphorylation in mitochondria **(A)** and ribosome submits **(B)** are mapped using KEGG pathway and labeled by red stars. **(C)** qPCR quantification of the relative gene expression of selected ribosome subunits or OXPHOS components. *Nono* housekeeping gene was used as the internal control for normalization. Student's *t-*test was applied to compare the value of PolydAdT or PolyIC to the Mock group, respectively. *p < 0.05, **p < 0.01, ***p < 0.001. *n* ≥ 3 biological replicates. Pathway maps in **(A,B)** are modified from KEGG pathway maps (with copyright permission for publication) using DAVID bioinformatics tool (36).

It is noticeable that multiple gene ontology or KEGG pathway terms related to mitochondria and ribosome were identified in the down-regulated genes (Figure 6B; Figure S3A). We examined the genes related to oxidative phosphorylation (OXPHOS) in mitochondria and structural components of ribosome (Figure 7). Strikingly, the vast majority of genes encoding for the complex I–V in the respiratory chain of mitochondria showed consistent down-regulation (Figure 7A; Figure S6A). Similar results were observed for genes encoding ribosomal proteins localizing to both small and large ribosomal subunits (Figure 7B; Figure S6B). qPCR analysis further confirmed the down-regulation of multiple ribosome subunit genes such as *Rps25, Rpl9, Mrpl18*,*Rpl36a*, and the OXPHOS genes such as *Sdhc* and *Sdhd* (Figure 7C). These results suggest that activation of antiviral immunity has a negative impact on mitochondria and ribosomes.

Collectively, we show evidence that antiviral immunity activation leads to the down-regulation of gene programs in a wide spectrum of general biological processes, cellular components, and pathways. It is important to point out that these biological processes, cellular components, and pathways affected were only significantly enriched in the down-regulated genes. In contrast, among the up-regulated genes we identified multiple pathways for antiviral or anti-microbial defense. Altogether, our findings demonstrate that the activation of antiviral immunity by viral mimics differentially affects the expression of sets of gene programs.

## Discussion

Synthetic viral genome analogs can activate the antiviral responses and induce the expression of type I interferon in mammalian cells of different species, such as mouse embryonic fibroblasts, human epithelial cells, rat hepatocyte, dog keratinocyte (28–31). In this study, we profiled the transcriptome changes of mouse embryonic fibroblast (MEFs) in response to viral mimic stimulation. Since the activation of antiviral responses by viral mimics seem to be highly conserved in different mammalian cells, mouse fibroblast can be a useful cell model to study the transcriptome changes by these viral analogs. We identified genes commonly up-regulated or down-regulated in response to dsDNA [poly (dA-dT)] and dsRNA [Poly (I:C)] viral mimics. The expression of more than 8,000 genes are affected by poly (dA-dT) or Poly (I:C) stimulation after 6 h. The vast majority of the affected genes shows similar trend of up-regulation or down-regulation in response to poly (dA-dT) and Poly (I:C) stimulation, indicating that dsDNA and dsRNA mimics activate or suppress similar cellular processes and signaling pathways. The overall extent of gene up-regulation is significantly higher compared to gene down-regulation. This basically reflects the fact that the antiviral immunity pathway leads to major gene induction events, such as the induction of type-I interferons due to the activation of IRF3 and IRF7 and the subsequent up-regulation of interferon-stimulated genes (8, 65).

For the analysis, we focused on the genes up-regulated or down-regulated by both poly (dA-dT) and Poly (I:C) stimulation, because they are more likely to represent the set of genes affected by both dsDNA and dsRNA viral infection. Biological processes such as defense response to virus, negative regulation of viral genome response and innate immune response are very significantly over-represented in the up-regulated genes, which demonstrates that that poly (dA-dT) and Poly (I:C) stimulation faithfully activates the antiviral immunity pathway and antiviral response genes in our data.

Recent studies have shown that there is a positive feedback up-regulation of several antiviral mediators such as RIG-I, STING, cGAS, and IRF1 after viral mimic or type-I interferon treatment (66–69). Consistently, our data identified these genes being up-regulated after viral mimic stimulation. More importantly, we show evidence that the activation of antiviral immunity leads to a widespread positive feedback regulation of multiple signaling pathways which presumably function to defend against viral or microbial infections. Among these pathways, we identified Toll-like receptor signaling pathway, cytosolic DNA sensing pathway, RIG-I-Like receptor signaling pathway, NOD-like receptor pathway. Detailed analysis demonstrates that this positive feedback enhances the expression of genes functioning at different steps of these pathways, including sensors, signal transducers and transcription factors. Collectively, our study not only supports the previously identified positive feedback induction of certain genes in the antiviral signaling pathway, but also extends the positive feedback regulation to many genes functioning at multiple steps of several antiviral or anti-microbial pathways. It has been shown that the up-regulation of cGAS and STING are dependent on type-I interferon (66, 67), while the induction of RIG-I gene seems to be interferon-independent and IRF3-dependent (68). It remains to be further determined for which group of genes the positive feedback up-regulation relies on the type-I interferon, and for which group of genes the up-regulation only requires the activation of IRFs.

Several recent studies revealed that viral infection can elicit strong DNA damage response in the host cells (45, 70, 71). These studies demonstrate that the DNA damage response signaling cascade is activated at posttranslational level upon viral infection, one of the primary targets being the phosphorylation of ATM/ATR kinase and H2AX. Our data add novel insights at the transcriptome level, showing that viral mimics can up-regulate a group of genes involved in DNA damage response and DNA repair. These genes can be induced by both dsDNA and dsRNA viral mimics. The exact role of DNA damage response pathway activation in viral infection may depend on the type of virus and the host cells. In some cases the activation of DNA damage response may confer antiviral effect (72, 73), while for certain virus it seems to be essential for the efficient viral genome replication in the host cells (74–76). These are interesting scenarios but the exact functional roles of the genes up-regulated by viral mimic stimulation need to be further clarified.

Another important finding is the identification of groups of helicase genes, nuclease genes and methyltransferase genes can be up-regulated upon the activation of antiviral immunity. Some of the genes are previously identified interferon-stimulated genes such as *Isg20, Rnasel, Ifih1, Ddx58* (77). Genes which have been recently shown to exhibit antiviral activity such as *Mov10* (51), *Helz2* (52), *Setdb2* (59), *Prmt6* (60) are also found in our list of up-regulated genes. For example, Helz2 mediates the suppression of Dengue Virus, and Prmt6 methyltrasnferase seems to inhibit the genome replication of HIV-1 (52, 60). Apart from those genes with known antiviral function, we report novel classes of genes being induced upon the activation of antiviral immunity, including multiple members of MCM (Minichromosome maintenance protein complex) family, DDX (DEAD-box helicases) family, DHX (DEAH-box helicases), histone methyltransferases, and RNA methyltransferases. Therefore, our data set provides a valuable resource for further characterizing the functional role of these seemingly novel players in the context of antiviral immunity.

The ubiquitin system is an important regulator in diverse cellular processes such as protein turnover, endocytosis, innate immunity and other signaling pathways (78–80). Viruses have a complex interplay with the ubiquitin-proteasome system of the host cells. On one hand, viruses can highjack the host ubiquitin system to facilitate entry into the host cell and replication (81, 82) or evade the antiviral immunity (83, 84). On the other hand, the ubiquitination of viral proteins by Trim22 and Trim32 for proteasome degradation can restrict viral replication (14, 15). Importantly, ubiquitination also regulates the antiviral immunity pathway. For example, the activation of antiviral sensor RIG-I depends on the ubiquitin (16), and Trim4, Trim25, and Rnf135 can, respectively ubiquitinate RIG-I to trigger its activation (17–19). Trim6, Trim56, and Herc6 can also potentiate antiviral immunity by targeting other antiviral effectors in the infection of vesicular stomatitis virus (VSV), pestivirus, and influenza A virus, respectively (20, 21, 64). In contrast, Trim29 can negatively regulates innate immune response by targeting RIG-I and STING (11, 22, 23). In line with these observations, our analysis identified *Herc6, Rnf135, Trim6, Trim25, Trim32* genes being significantly up-regulated upon the stimulation of viral mimics, while *Trim29* was not induced. We expect that the up-regulation of these genes positively enhances the antiviral immunity, adding another layer of positive feedback regulation. Moreover, there are many other ubiquitin-transferase or ligase genes, including multiple members of Trim and Rnf gene families. Although further analysis is required to understand their specific roles, the enhanced expression of ubiquitin-transferases or ligases upon the activation of antiviral immunity suggests an augmented ubiquitination activity upon viral infection and a possible direct involvement in antiviral defense.

In addition to the identification of novel groups of genes being induced, our study also shows that a broad spectrum of gene programs related to general biological processes, cellular components, and pathway is down-regulated upon activation of antiviral immunity. We identified gene programs involved in multiple metabolic pathways, biological processes such as cell cycle, protein translation and processing, cellular components such as cytoskeleton, mitochondria, and ribosome.

Previous studies found that viral infection can lead to suppression of certain metabolic pathways, such as fatty acid synthesis and sterol synthesis (85, 86). A recent study also showed that viral analogs down-regulated genes involved in metabolic process (87). There is also evidence that viral infection down-regulates genes involved in ribosome biogenesis and cytoskeleton regulation (88, 89). By identifying novel gene programs that are down-regulated, our study provides a more complete picture of the cellular processes and components negatively affected upon viral infection.

Viral genome analogs such as poly(dA-dT) and poly(I:C) serve as a simple and efficient way to activate the antiviral immunity in a variety of cell types. However, we have to point out that proteins encoded by different virus can also modulate the activation of innate immunity (90). Therefore, the influence of associated viral proteins is not captured in our data. Since the proteins expressed by different classes of virus vary, the degrees and duration of antiviral immunity elicited by different virus are expected to be different. Nevertheless, the understanding of transcriptome changes induced by viral genome analogs reveals novel groups of genes being up-regulated, which can be potential candidates for regulators or effectors in antiviral immune response.

In summary, our study provides valuable information about the transcriptome changes upon antiviral immunity activation. Our data revealed that gene programs associated with a wide range of general cellular processes and components are down-regulated, indicative of the cellular activities that are potentially negatively affected upon viral infection. We also identified novel groups of genes being up-regulated, altogether providing a useful resource for mining new antiviral effectors and characterizing their functions.

## Author Contributions

XX and PP designed the research; XX performed the research; XX, P-SL, and PP analyzed the data and wrote the paper. All authors read and approved the final manuscript.

### Conflict of Interest Statement

The authors declare that the research was conducted in the absence of any commercial or financial relationships that could be construed as a potential conflict of interest.

## Supplementary Material

Table S1 DNA sequence of primers used for qPCR analysis.

Table S2 Summary of the Gene Ontology and KEGG pathway analysis. 1. Biological processes; 2. Cellular components; 3. Molecular functions; and 4. KEGG pathways that are over-represented in the commonly up-regulated or down-regulated genes are shown respectively. The GO terms or KEGG pathway term with at least 20 gene counts, fold enrichment ≥, *P* < 0.01 were considered to be significantly over-represented in each gene list.

Figure S1 Heat map showing the relative expression levels of up-regulated genes by viral mimic stimulation involved in defense response to virus. Scale bar: log2 (relative expression level).

Figure S2 Genes up-regulated are mapped to the RIG-I like receptor pathway **(A)** and TNF signaling pathway **(B)**, which are labeled by red stars.

Figure S3 Summary of KEGG pathways over-represented in the down-regulated gene list is shown in **(A)**. **(B)** Down-regulated genes involved in fatty acid metabolism is mapped to the KEGG pathway. The down-regulated genes responsible for each enzymatic steps are labeled with red star.

Figure S4 Down-regulated genes involved in cell cycle **(A)**, citrate cycle **(B)**, and protein processing in endoplasmic reticulum **(C)** is mapped to the KEGG pathway. The down-regulated genes responsible for each enzymatic steps are labeled with red star.

Figure S5 Heat map showing the relative expression levels of down-regulated genes by viral mimic stimulation related to cell cycle **(A)**, citrate cycle **(B)**, and protein processing in endoplasmic reticulum **(C)**. Scale bar: log2 (relative expression level).

Figure S6 Heat map showing the relative expression levels of down-regulated genes by viral mimic stimulation related to oxidative phosphorylation **(A)** and ribosome submits **(B)**. Scale bar: log2 (relative expression level).
