# Supplementary figures and images for: Analysis of Global Transcriptome Change in Mouse Embryonic Fibroblasts After dsDNA and dsRNA Viral Mimic Stimulation
Source: Front Immunol. 2019 Apr 17;10:836. doi: 10.3389/fimmu.2019.00836 (PMC6478819; doi:10.3389/fimmu.2019.00836)

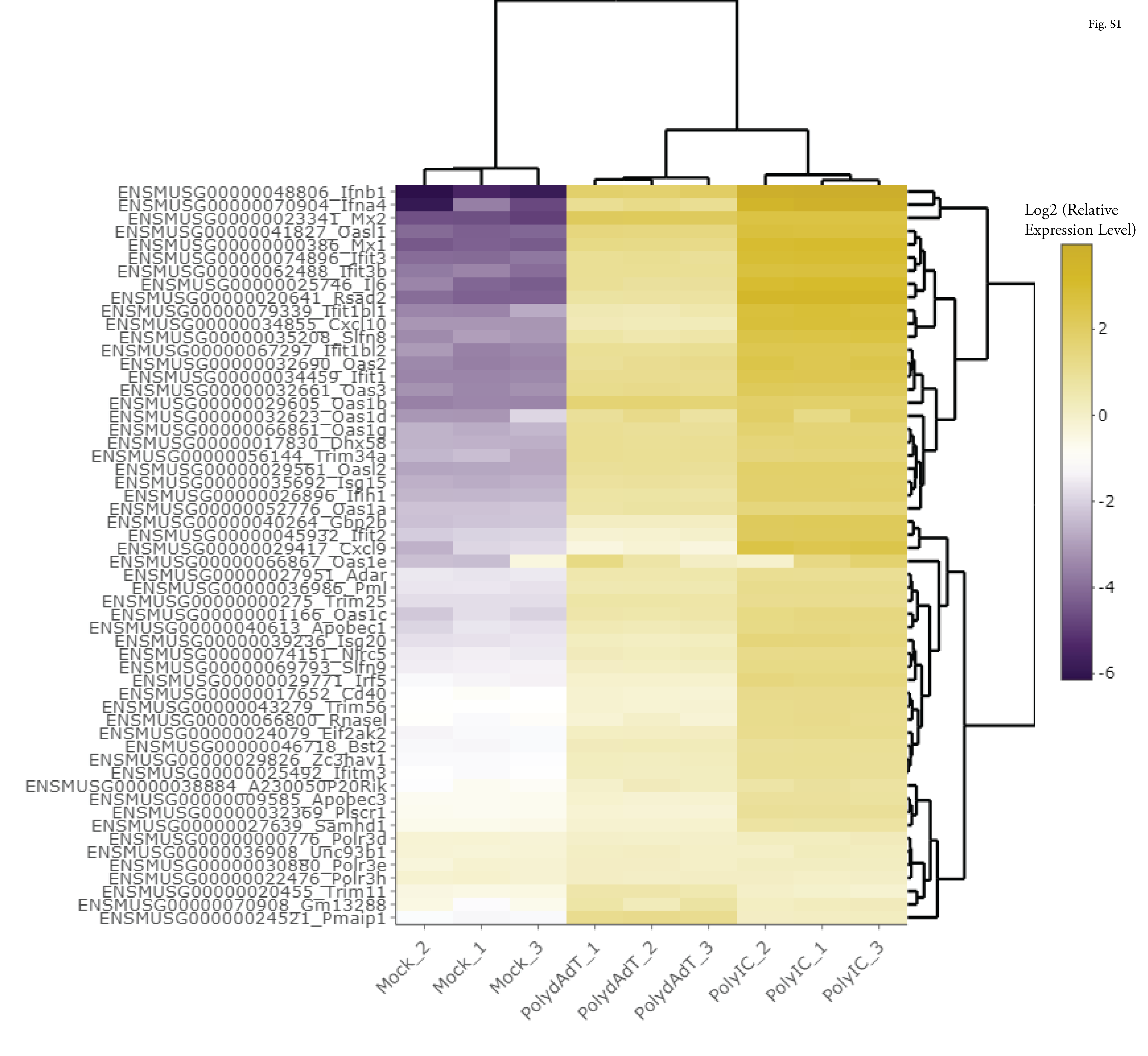

Supplement: Figure S1 — Heat map showing the relative expression levels of up-regulated genes by viral mimic stimulation involved in defense response to virus. Scale bar: log2 (relative expression level). [file Image_1.TIF]

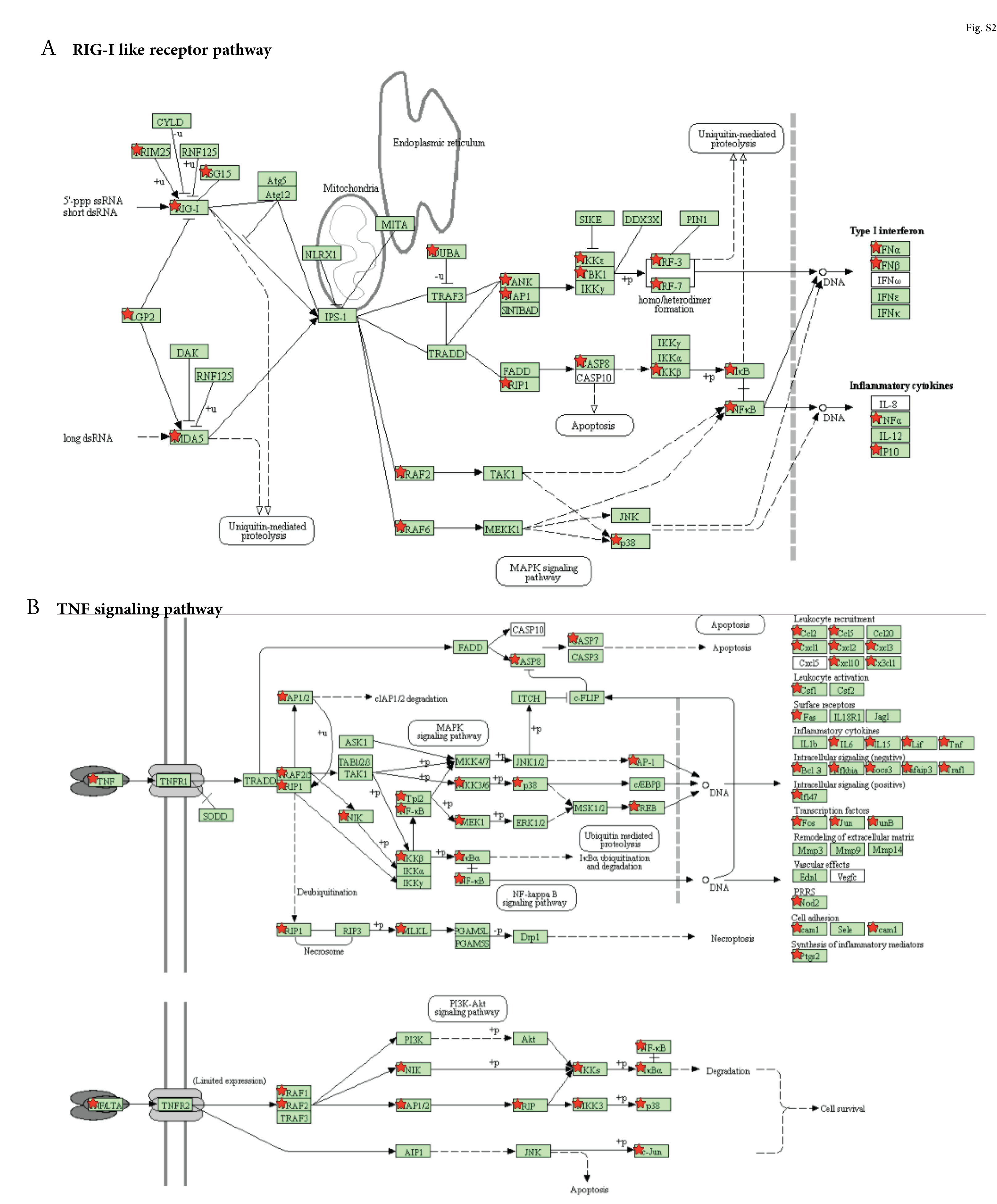

Supplement: Figure S2 — Genes up-regulated are mapped to the RIG-I like receptor pathway (A) and TNF signaling pathway (B), which are labeled by red stars. [file Image_2.TIF]

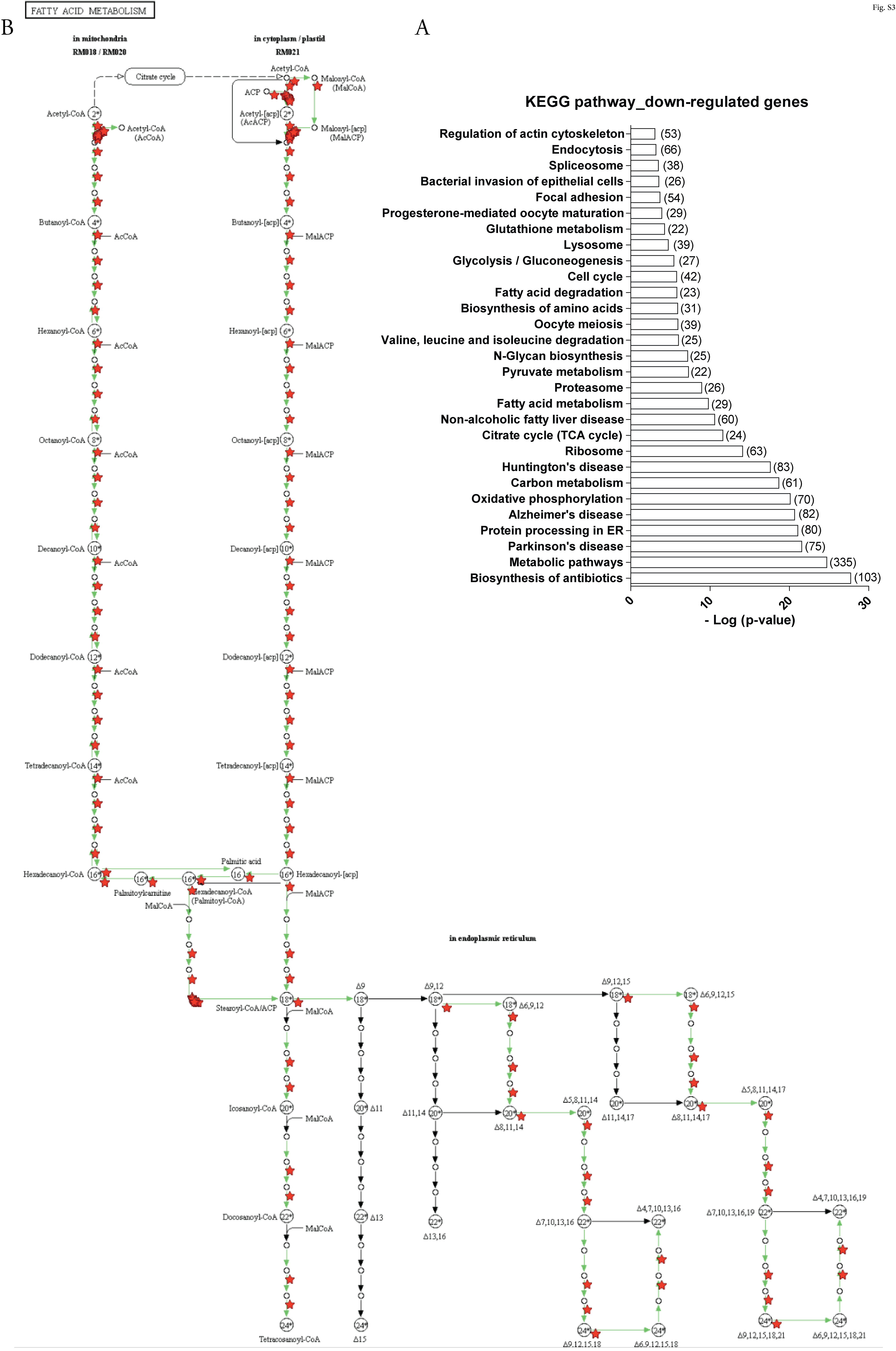

Supplement: Figure S3 — Summary of KEGG pathways over-represented in the down-regulated gene list is shown in (A). (B) Down-regulated genes involved in fatty acid metabolism is mapped to the KEGG pathway. The down-regulated genes responsible for each enzymatic steps are labeled with red star. [file Image_3.TIF]

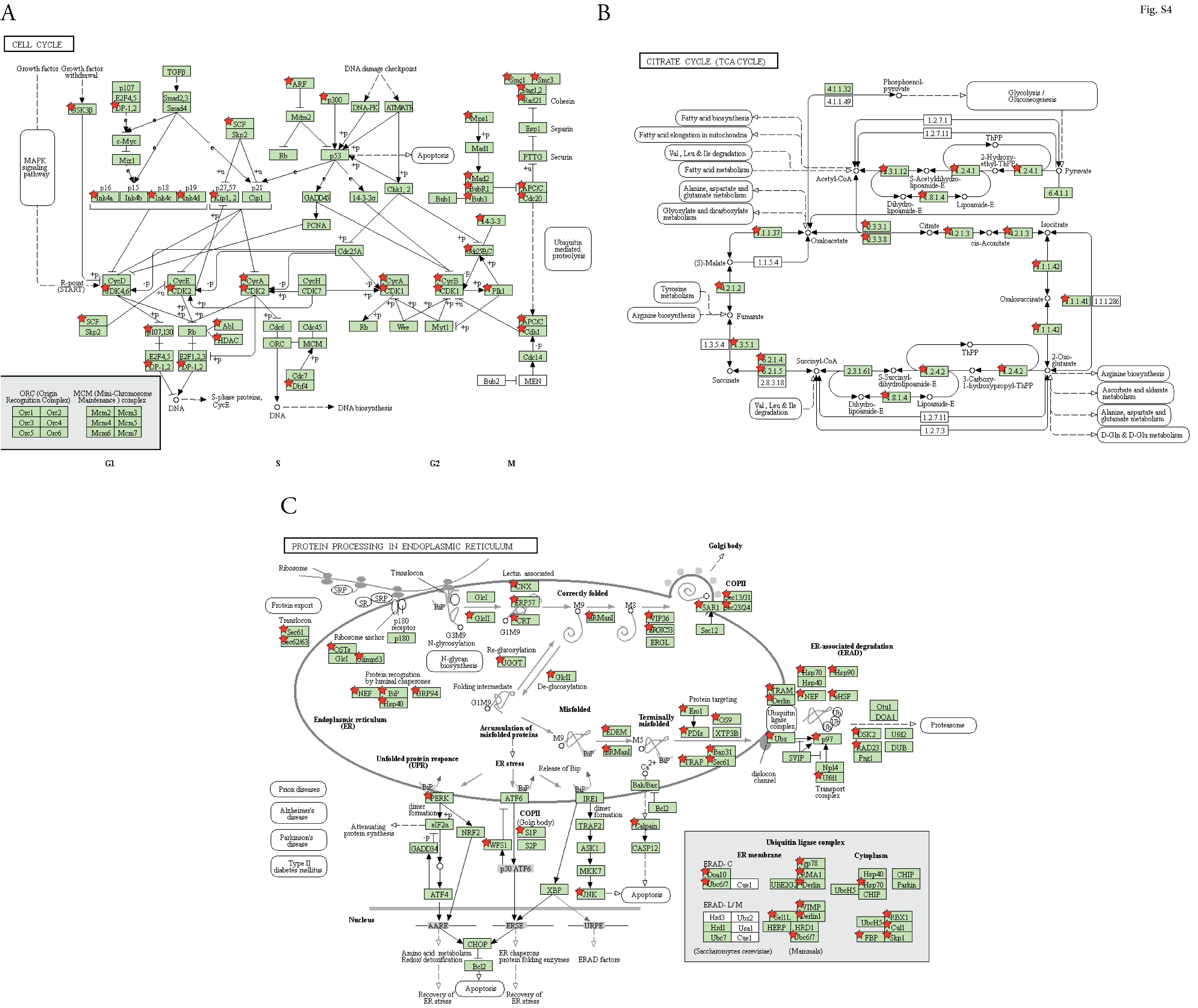

Supplement: Figure S4 — Down-regulated genes involved in cell cycle (A), citrate cycle (B), and protein processing in endoplasmic reticulum (C) is mapped to the KEGG pathway. The down-regulated genes responsible for each enzymatic steps are labeled with red star. [file Image_4.TIF]

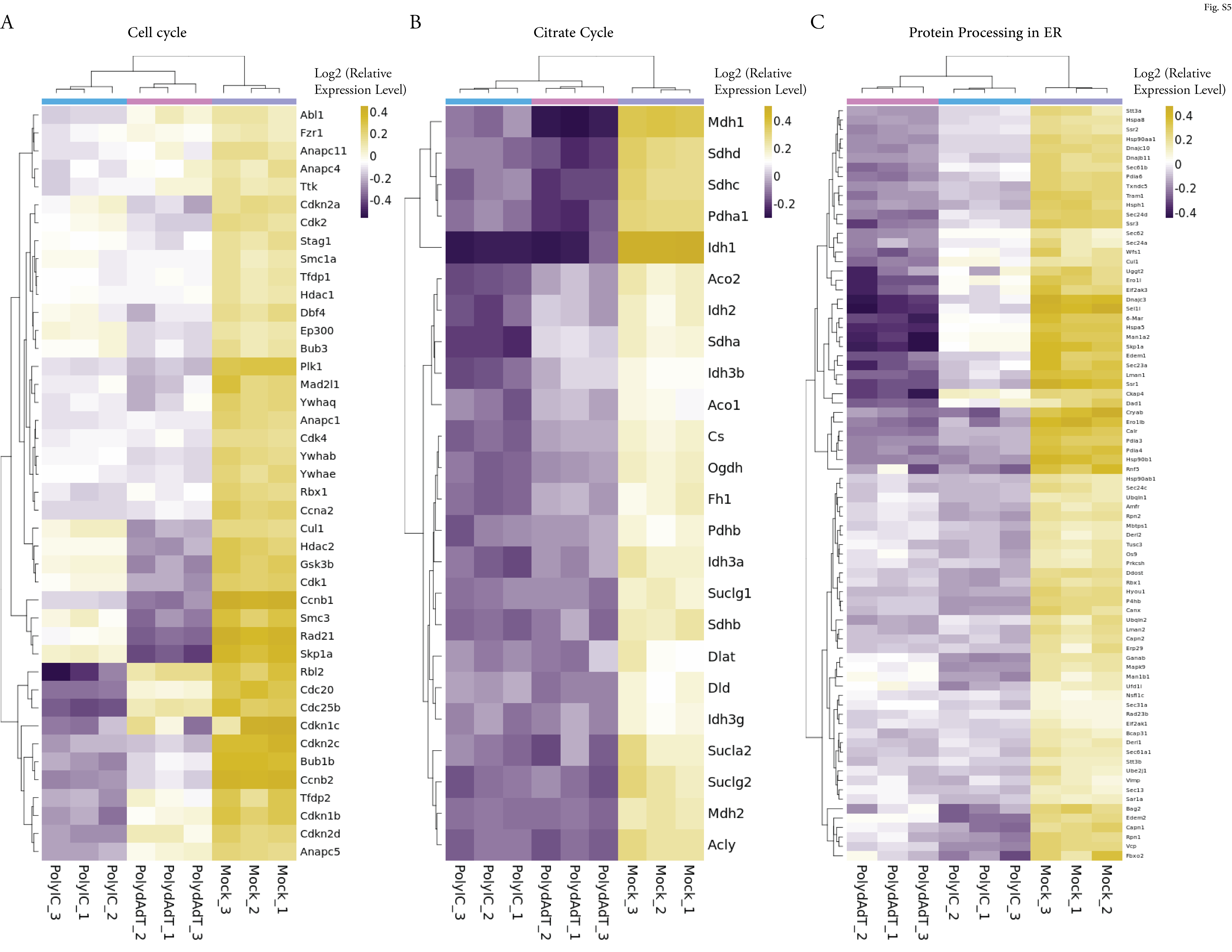

Supplement: Figure S5 — Heat map showing the relative expression levels of down-regulated genes by viral mimic stimulation related to cell cycle (A), citrate cycle (B), and protein processing in endoplasmic reticulum (C). Scale bar: log2 (relative expression level). [file Image_5.TIF]

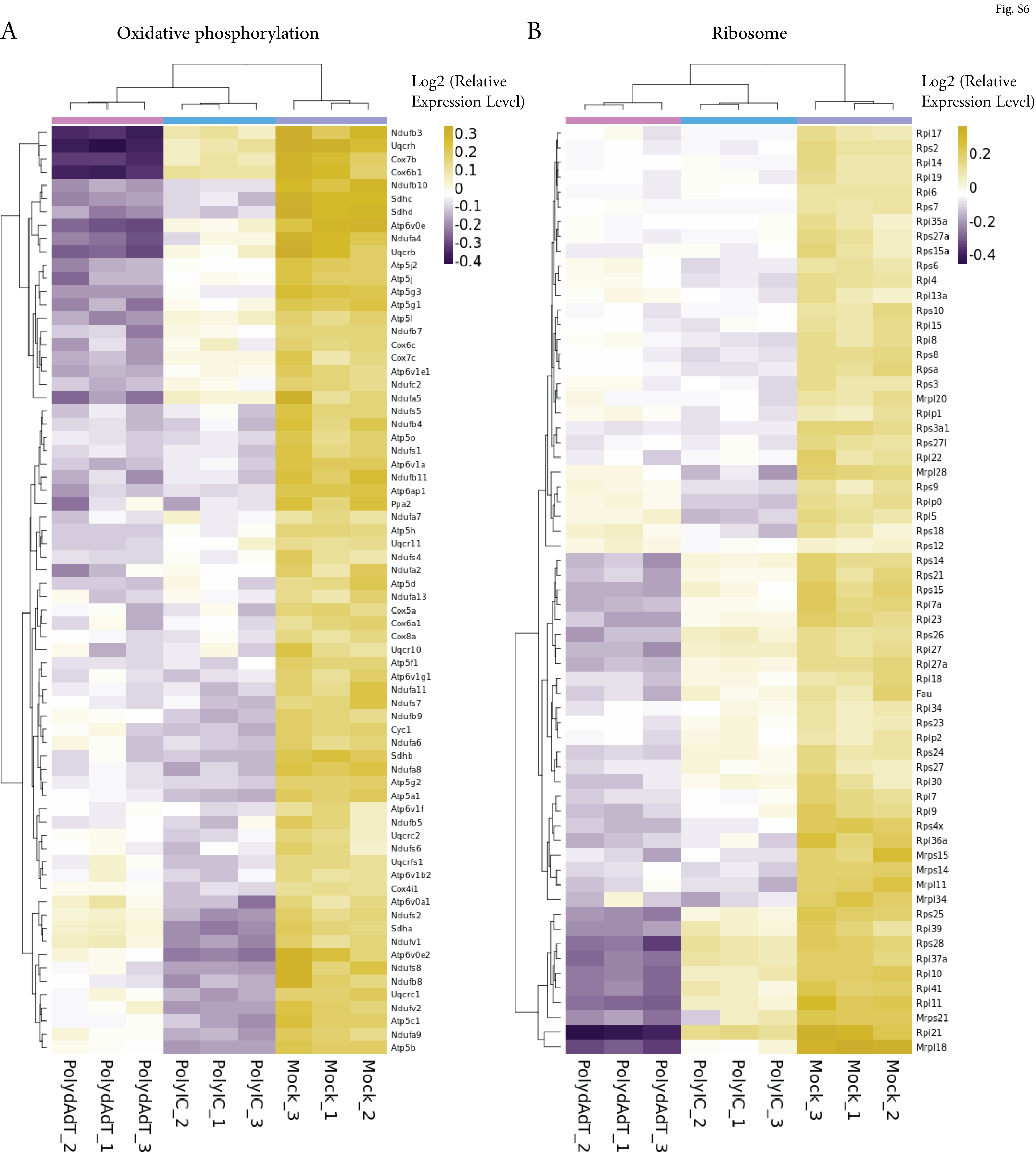

Supplement: Figure S6 — Heat map showing the relative expression levels of down-regulated genes by viral mimic stimulation related to oxidative phosphorylation (A) and ribosome submits (B). Scale bar: log2 (relative expression level). [file Image_6.TIF]
